# Sonographic diagnosis of carpal tunnel syndrome: a study in 200 hospital workers*

**DOI:** 10.1590/0100-3984.2014.0069

**Published:** 2015

**Authors:** Adham do Amaral e Castro, Thelma Larocca Skare, Paulo Afonso Nunes Nassif, Alexandre Kaue Sakuma, Wagner Haese Barros

**Affiliations:** 1 Master, MD, Radiologist, Postgraduate student of Ultrasonography, Computed Tomography and Magnetic Resonance Imaging at Hospital Israelita Albert Einstein, São Paulo, Part Time Dedication, Fellow PhD degree, Instituto de Pesquisas Médicas, Faculdade Evangélica do Paraná – Hospital Universitário Evangélico de Curitiba, Curitiba, PR, Brazil.; 2 PhD, MD, Rheumatologist, Head of Rheumatology Service, Hospital Universitário Evangélico de Curitiba, Full Professor of Rheumatology, Faculdade Evangélica do Paraná, Curitiba, PR, Brazil.; 3 PhD, MD, Digestive System Surgeon, Head of the Service of Bariatric and Metabolic Surgery, Hospital Universitário Evangélico de Curitiba, Associate Professor of Traumatology, Surgical Practice II and Scientific Methodology at Faculdade Evangélica do Paraná, Curitiba, PR, Brazil.; 4 MD, Scientific Research student of Instituto de Pesquisas Médicas da Faculdade Evangélica do Paraná – Hospital Universitário Evangélico de Curitiba, Curitiba, PR, Brasil.; 5 MD, Radiologist, Trainee in Locomotor System Radiology at Hospital Alemão Oswaldo Cruz, São Paulo, SP, Brazil.

**Keywords:** Carpal tunnel syndrome, Ultrasonography, Median nerve area, Hand pain, Hand paresthesia

## Abstract

**Objective:**

To describe the prevalence of carpal tunnel syndrome in a sample of 200 healthy hospital workers, establishing the respective epidemiological associations.

**Materials and Methods:**

Two hundred individuals were submitted to wrist ultrasonography to measure the median nerve area. They were questioned and examined for epidemiological data, body mass index, carpal tunnel syndrome signs and symptoms, and submitted to the Boston carpal tunnel questionnaire (BCTQ) to evaluate the carpal tunnel syndrome severity. A median nerve area ≥ 9 mm^2^ was considered to be diagnostic of carpal tunnel syndrome.

**Results:**

Carpal tunnel syndrome was diagnosed by ultrasonography in 34% of the sample. It was observed the association of carpal tunnel syndrome with age (*p* < 0.0001), paresthesia (*p* < 0.0001), Tinel’s test (*p* < 0.0001), Phalen’s test (*p* < 0.0001), BCTQ score (*p* < 0.0001), and years of formal education (*p* < 0.0001). Years of formal education was the only variable identified as an independent risk factor for carpal tunnel syndrome (95% CI = 1.03 to 1.24).

**Conclusion:**

The prevalence of carpal tunnel syndrome in a population of hospital workers was of 34%. The number of years of formal education was the only independent risk factor for carpal tunnel syndrome.

## Abstract (pt)

**Objetivo:**

Descrever a prevalência da síndrome do túnel do carpo em amostra de 200 trabalhadores hospitalares sem comorbidades conhecidas e suas associações epidemiológicas.

**Materiais e Métodos:**

Duzentos indivíduos foram submetidos a ultrassonografia de punhos para mensuração da área da secção transversa do nervo mediano. Eles foram questionados e examinados sobre dados epidemiológicos, índice de massa corporal, sinais e sintomas da síndrome do túnel do carpo e submetidos ao *Boston carpal tunnel questionnaire* (BCTQ) para avaliação da gravidade da síndrome do túnel do carpo. A área da secção transversa do nervo mediano ≥ 9 mm^2^ foi considerada diagnóstica de síndrome do túnel do carpo.

**Resultados:**

A síndrome do túnel do carpo foi diagnosticada por ultrassonografia em 34% da amostra estudada. Foi observada associação desta síndrome com idade (*p* < 0,0001), parestesia (*p* < 0,0001), teste de Tinel (*p* < 0,0001), teste de Phalen (*p* < 0,0001), escore do BCTQ (*p* < 0,0001) e anos de educação formal (*p* < 0,0001). Anos de educação formal foi a única variável identificada como fator de risco independente para síndrome do túnel do carpo (IC 95%: 1,03 a 1,24).

**Conclusão:**

A prevalência da síndrome do túnel do carpo na amostra de trabalhadores hospitalares estudada foi 34%. O número de anos de educação formal foi o único fator de risco independente para síndrome do túnel do carpo.

## INTRODUCTION

Carpal tunnel syndrome (CTS) results from compression of the median nerve at the level of the carpal tunnel. It is the most frequent compressive neuropathy, with prevalence in the general population of 9.2% in women and 6% in men^(1)^. According to Ono et al.^(2)^, CTS is associated with the second longest average time away from work and its cost is estimated to be US$30,000 per worker in the United States of America.

Obesity, pregnancy, diabetes mellitus, hypothyroidism, among other conditions, are associated with CTS^(3)^. The occupational factor plays a relevant role in the development of this syndrome, particularly in case of tasks involving increased vibration, use of great strength and repetitive strain with the hands^(4)^.

The CTS diagnosis is based on clinical criteria, but it may be supplemented by tests such as electroneuromyography and imaging methods such as ultrasonography (US) and magnetic resonance imaging (MRI)^(5)^. US takes less time to be performed, causes less discomfort to the patient and may be considered to be a more cost-effective strategy as a first-line method to confirm a clinical suspicion of CTS^(6)^. The measurement of the median nerve area (MNA) is the most important diagnostic criterion for CTS^(7)^, and 9 mm^2^ is the most accurate cutoff point^(8)^.

This study aimed to describe the prevalence of CTS diagnosed by US and to establish its epidemiological associations in 200 healthy volunteers.

## MATERIALS AND METHODS

Two hundred hospital workers with no self-reported known comorbidity (35 men and 165 women) were invited to participate in the study. Age above 18 was the inclusion criterion. Exclusion criteria were the following: pregnancy; untreated hypothyroidism; chronic renal failure under dialysis; history of repetitive strain injury; recent trauma affecting upper limbs and any form of arthritis; conditions that might be associated with median nerve neuropathy. After approval by the Committee for Ethics in Research of the institution and signing on a term of free and informed consent by the participants, all of them completed a Katz diagram for pain and paresthesia in the region of the median nerve^(9)^. The physical examination included measurement of height and weight for calculation of the body mass index (BMI)^(10)^ and hands examination by means of the Tinel's and Phalen's tests^(9)^. The Tinel's test was performed by repeatedly tapping on the median nerve of the wrist for 4-6 times^(9)^. The presence or absence of pain irradiation or paresthesia in the median nerve distribution was recorded. The Phalen's test was performed by asking the patient to maintain complete palmar flexion of the wrist, with extended elbow and pronated forearm. The Phalen's test was considered to be positive upon symptoms reproduction in up to one minute^(9)^.

The validated Brazilian version of the Boston carpal tunnel questionnaire (BCTQ) was utilized for a specific evaluation of the CTS symptoms severity^(11)^. Higher BCTQ scores are associated with higher degree of damages caused by CTS^(12)^.

**Figure 1:**
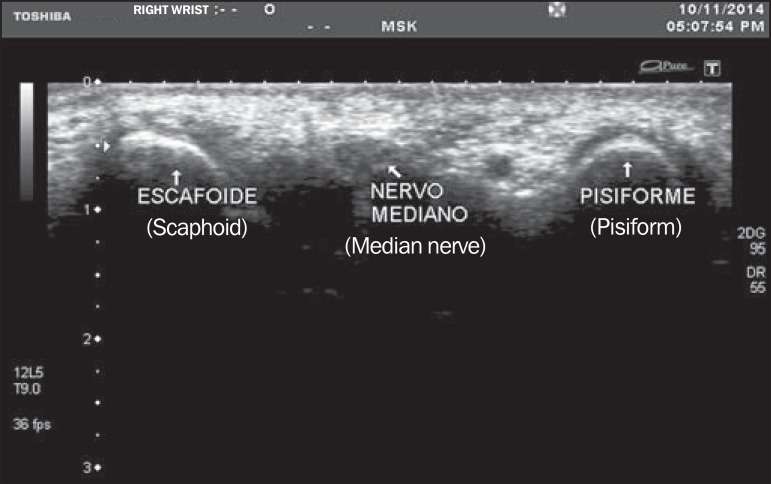
Median nerve visualized in the region of the pisiform and scaphoid bones.

**Figure 2:**
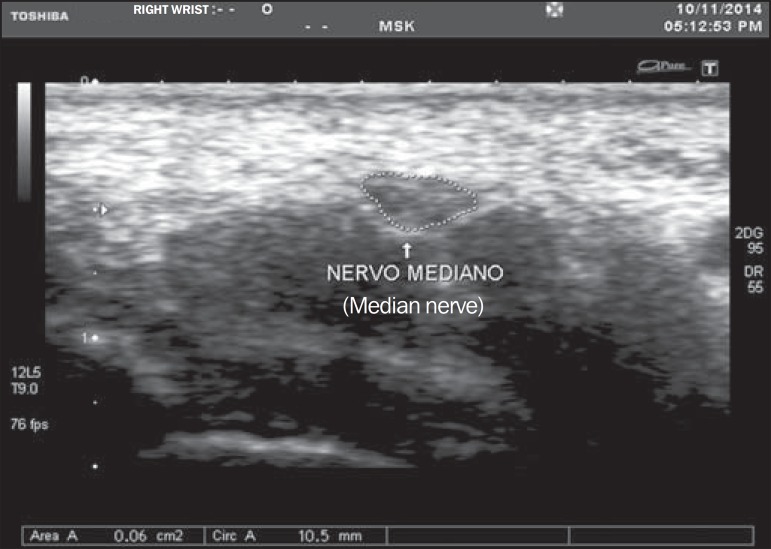
Cross section of the normal (delineated) median nerve area.

The MNA was measured by US with a Xario^®^ Toshiba apparatus and a linear 12 MHz multifrequency transducer over the distal palmar surface of the wrist (at the level of the proximal flexion fold). The pisiform bone knobs and the scaphoid tubercle were identified by palpation (Figure 1). A single sonographer who was blind for the patients' clinical data performed the measurements. The patients were seated, with the arm in supination on a table, with the wrist in neutral position and the semiflexed fingers at rest. The MNA was automatically calculated by the US apparatus on the basis of a continuous line drawn by the sonographer around the nerve margin (Figure 2) defined as the external margin of the hypoechoic nerve fascicles and the interior of the hyperechoic nerve sheath^(13)^. A MNA ≥ 9 mm^2^ was considered to be diagnostic for CTS^(7)^. As both hands were evaluated, the value considered for statistical purposes was the one regarding the hand with larger MNA.

The data were collected and organized on frequency and contingency tables and the sample distribution was analyzed by the Kolmogorov-Smirnov test.

The central tendency was expressed as median values as a function of the non-parametric sampling. The chi-square test (for nominal data) and the Mann-Whitney test (for numerical data) were utilized for association studies. Also, the variables involved in the cause of CTS with significance in the univariate analysis were studied by means of logistic regression to assess their independence. The adopted significance level was 5%. The calculation was performed with the aid of the Medcalc^®^ 10.0 software.

## RESULTS

The present study sample included 35 men and 165 women with mean age corresponding to 40.0 years (range = 18-74 years; interquartile range = 27.0-49.0 years). In the sample, 39/200 (19.5%) of the participants self-reported to be Afrodescendants; 156/200 (78%), Caucasian; and 5/200 (2.5%) self-reported to be Oriental. As regards work activities, 142/200 (71%) individuals had manual occupations and 58/200 (29%), non-manual occupations.

Diagnosis of CTS by US was performed in 34% of the sample. The CTS epidemiological and clinical associations are shown on Table 1.

**Figure 3:**
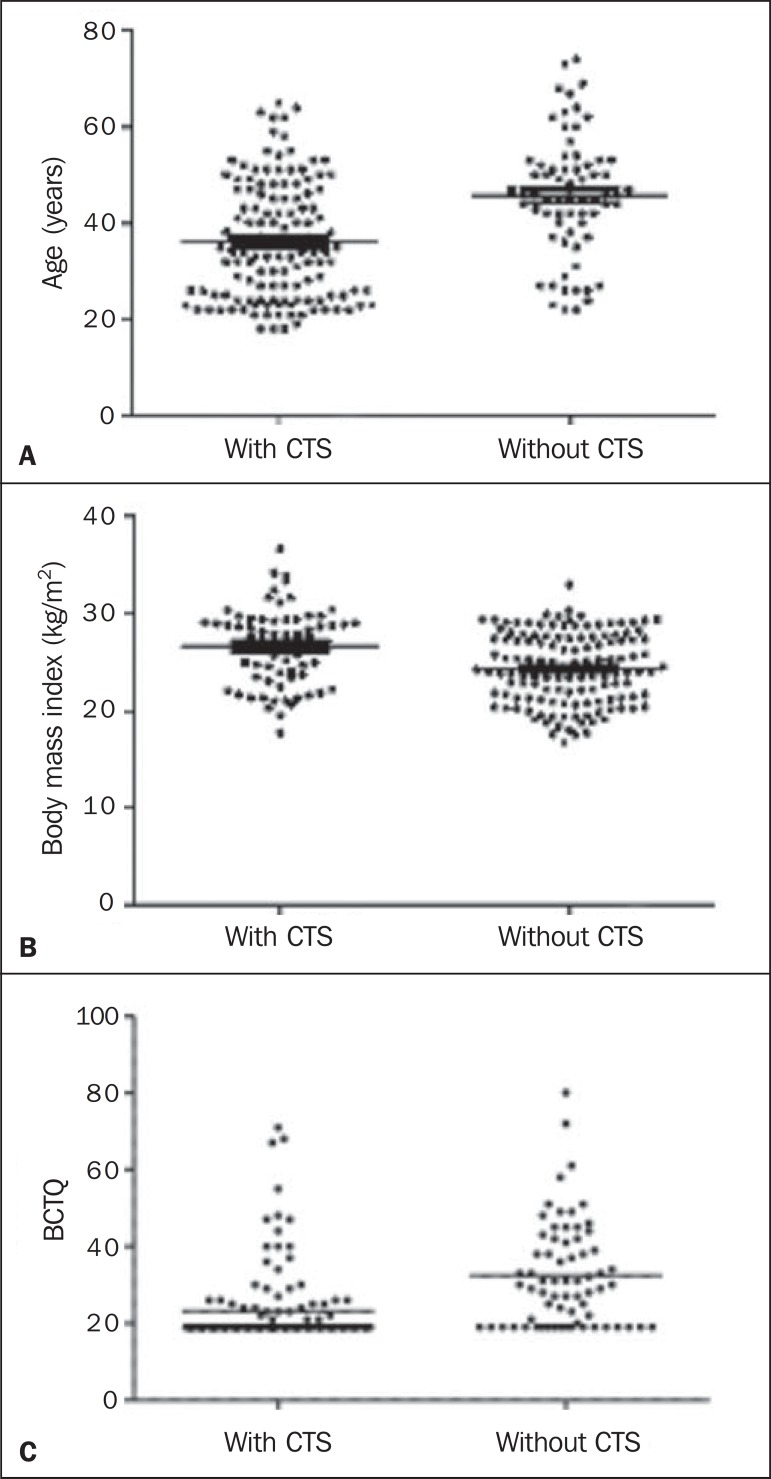
Association between CTS and age (**A**), Body mass index (**B**) and BCTQ score (**C**) in 200 individuals.

Associations between CTS and age, BMI, number of years of formal education and BCTQ score were observed (Figure 3).

**Table 1:** Clinical and epidemiological association of CTS in a sample of 200 volunteers without any comorbidity.

	With CTS (*n* = 68)	Without CTS (*n* = 132)	*p*
Sex	15 men; 53 women	22 men; 110 women	0.35*
Age (years)	22.0 to 74.0 (median, 46.0)	18.0 to 65.0 (median, 34.0)	< 0.0001
Body mass index	17.7 to 36.68 (median, 27.62)	16.72 to 33.05 (median, 24.35)	0.0002†
Racial group	Afrodescendants: 14; Caucasian: 53; Asian: 1	Afrodescendants: 24; Caucasian: 104; Asian: 4	0.74*
Occupation	Manual: 56/68 (82.3%); non-manual: 16/68 (23.5%)	Manual: 86/132 (65.1%); non-manual: 46/132 (34.8%)	< 0.0001†
Years of formal education	0 to 16 (median, 8)	2.0 to 20.0 (median, 11.0)	< 0.0001†
Paresthesia§	44/68 (64.7%)	21/132 (15.9%)	< 0.0001*
Pain§	37/68 (54.4%)	27/132 (20.4%)	< 0.0001*
Positive Tinel's test	42/68 (61.7%)	21/132 (15.9%)	< 0.0001*
Positive Phalen's test	40/68 (58.8%)	20/132 (15.1%)	< 0.0001*
BCTQ	19.0 to 80.0 (median, 30.0)	19.0 to 71.0 (median, 19.0)	< 0.0001†

* Chi- square test;

† Mann-Whitney test;

§ According to Katz diagram.

Logistic regression analysis demonstrated the variable "years of formal education" as an independent risk factor for CTS (Table 2).

**Table 2:** Logistic regression analysis of variables associated with CTS.

Variable	*Odds ratio*	CI 95%
Years of formal education	1.13	1.03-1.24
Body mass index	1.00	0.92-1.08
Age	1.00	0.97-1.03
Manual occupation	1.41	0.64-3.11

CI 95%, confidence interval 95%.

## DISCUSSION

In the present study, the authors found a high CTS prevalence (34%) in individuals who had never been previously diagnosed with this disease. Such a high prevalence, in contrast to the prevalence of 9.2% in women and 6% in men in the general population^(1)^, could be attributed to the marked presence of intensive labourers in the sample together with the direct approach by US scan, possibly resulting in an increase in the number of diagnoses of subclinical cases or even of false-positive diagnoses, considering that no other diagnostic approach was performed and the sonographic measurement was the only criterion utilized.

CTS is a very frequently found condition, resulting not only in impaired quality of life, but also in a significant financial cost to the health system^(6)^. The BCTQ is a tool capable of assessing the CTS severity with high validity, reliability and response capacity, demonstrating the impact of this condition on the patients' lives^(12)^. In the present study, the BCTQ scores were significantly higher in patients diagnosed with CTS at US.

Currently, the utility of US in rheumatology and orthopedics clinics is increasing, and one can consider that the wide utilization and applicability of the method in the context of CTS inclusive, is transforming the clinical practice in these specialties^(14)^. US is an imaging modality that can be considered to be a first-line diagnostic tool for CTS due to its noninvasiveness, wide availability and accuracy as compared with electroneuromyography^(15)^. Additionally, in the hand of a specialist, the method presents a good cost-benefit ratio^(6)^ when utilized as a screening tool in a population at risk such as that involved in manual occupations^(16)^. In the present study, US was utilized as an instrument of screening for CTS in a population of hospital workers presenting without any known comorbidity, and, among the individuals diagnosed with CTS by US, the authors observed a significant association with the classical clinical symptoms (pain and paresthesia in the median nerve area) and CTS signs such as Tinel's and Phalen's.

Activities associated with CTS include those involving prolonged, marked wrist flexion or extension, repetitive use of the flexor muscles and exposure to vibration^(17)^. In the present sample, despite the absence of details about the positioning of the hands during the work, the patients involved in manual occupations (such as nurses, hygiene workers and radiology technicians) presented higher CTS indices than non-manual workers (such as physicians and psychologists).

In a meta analysis on the US accuracy in the diagnosis of CTS, Carvalho et al.^(7)^ observed that the most relevant criterion for the US diagnosis was the calculation of the MNA with a cutoff point between 9 and 10 mm^2^. These authors have concluded that US can be utilized in the daily clinical practice as a first-line method in the diagnosis of CTS, with a level of evidence 1b. Although this is a controversial theme in the literature, the meta-analysis developed by Tai et al.^(8)^ indicates that the most accurate cutoff point for CTS is 9 mm^2^ - the value adopted in the present study. Among other criteria which could be utilized in the diagnosis of CTS, the measurement of the MNA at two different sites is highlighted, with calculation of the difference between the values^(18)^. This data may increase the accuracy in the diagnosis of CTS, as demonstrated by Klauser et al.^(18)^, but the present study did not contemplate such an approach.

Other clinical risk factors related to the CTS physiopathology include: pregnancy, menopause, obesity, renal failure, hypothyroidism, use of oral contraceptive drugs, congestive heart failure, local tumors and tumor-like lesions such as distal radius fracture, wrist arthritis, diabetes, alcoholism, vitamin toxicity or deficiency and exposure to toxins^(17)^. In the present study sample, age and BMI were higher in patients with CTS (*p* < 0.0001) for both variables. In the study developed by Komurcu et al., age and BMI not only were more prevalent in patients with CTS, but also were related to greater CTS severity^(19)^. Such authors utilized electroneuromyography to evaluate the CTS severity in already clinically diagnosed patients.

In spite of the fact that the present study has observed association between CTS and age, BMI, manual occupation and years of formal education in the univariate analysis, as the logistic regression analysis was performed, years of formal education was the only variable found as an independent risk factor for CTS, revealing the huge relevance of this aspects in the whole life of an individual. The present study has just considered the patient's report on years of formal education. A more comprehensive analysis might reveal a more relevant role of this variable for the development of CTS, since education may be associated with all the aspects in an individual's life, from feeding habits and BMI to his/ her position and insertion in the labor market. A sociological approach is beyond the scope of the present study, and should remain as a suggestion for further studies, considering the great relevance of this aspect contribution to the individuals' health, including the presence of CTS.

## CONCLUSION

In the present study, the prevalence of CTS was of 34%. The diagnosis of such a condition was associated with age, BMI, manual occupation and years of formal education at univariate analysis. Logistic regression revealed that only "years of formal education" remained as an independent risk factor for development of CTS.
